# Genetic variant of *SPARC* gene and its association with growth traits in Chinese cattle

**DOI:** 10.5194/aab-63-31-2020

**Published:** 2020-01-30

**Authors:** Danyang Zhang, Jiawei Xu, Peng Yang, Yifan Wen, Hua He, Jiaxiao Li, Juntong Liang, Yining Zheng, Zijing Zhang, Xianwei Wang, Xiang Yu, Eryao Wang, Chuzhao Lei, Hong Chen, Yongzhen Huang

**Affiliations:** 1 College of Animal Science and Technology, Northwest A&F University, Yangling, Shaanxi, 712100, People's Republic of China; 2 College of Veterinary Medicine, Northwest A&F University, Yangling, Shaanxi, 712100, People's Republic of China; 3 Institute of Animal Husbandry and Veterinary Science, Henan Academy of Agricultural Sciences, Zhengzhou, Henan, 45002, People's Republic of China; 4 Henan Provincial Animal Husbandry General Station, Zhengzhou, Henan, 450008, People's Republic of China; 5 Henan Animal Health Supervision Institute, Zhengzhou, Henan, 450003, People's Republic of China

## Abstract

SPARC is a cysteine-rich acidic secreted protein. It is a non-collagen component of bone, which is widely distributed in humans and animals and plays an important role. SPARC has been found in a variety of human cancers (breast cancer, stomach cancer, ovarian cancer, etc.) and diabetes-related research. Especially the muscle and fat metabolism are closely related. In this study, we used a DNA pool to detect a new SNP site (g.12454T > C). A total of 616 samples of four breeds of Qinchuan cattle (QC, n=176), Xianan cattle (XN, n=160), Pinan cattle (PN, n=136) and Jiaxian cattle (JX, n=144) were analyzed and identified with ARMS-PCR. In addition, we correlated SNP with growth traits and showed significant correlation with growth traits such as rump length, hip width, and body length (p<0.05). Moreover, we tested the *SPARC* gene expression level in different tissues belonging to XN adult cattle (n=3) and found its high expression in muscle tissues (relative to the kidney). Further, we found the SNP is able to increase the *SPARC* expression level in skeletal muscle (n=12). According to statistical data, this SNP site may be applied to a molecular marker of an early marker-assisted selection for early growth of beef cattle.

## 1 Introduction

The single nucleotide polymorphism (SNP) is the one nucleotide variation in the DNA sequence which will produce many polymorphisms in the genome of animals. It involves the transformation or transversion of a single nucleotide, which occurs in the sequence of the encoded protein or in the sequence of introns and intergenic regions (Dang et al., 2014). The SNP which happens in the CDS region will change and affect the protein if the mutation is the missense type. The other situation is the SNP happens in the CDS region, but it does not revise the coding of genes, which we call the synonymous mutation. The reason is the degeneracy of the codon. However, whatever the type of SNP, it will cause an impact of biological processes and sometimes it will cause phenotypic variation (Moravčíková et al., 2018; Carignano et al., 2018; Nakajima et al., 2018).

There are many methods of SNP genotype classification, such as RFLP, introduction mutation and direct sequencing. Moreover, the tetra-primer application refractory mutation system PCR (T-ARMS-PCR) is used as a low-cost, rapid genotyping assay (Hamajima et al., 2000).

The *SPARC* (secreted protein acidic and rich in cysteine) gene, which was first extracted by Termine et al. (1981), is a cysteine-rich acidic secreted protein, which is called the osteonectin or basement membrane 40 protein (Workman and Bradshaw, 2007). According to many current studies, the expression of SPARC protein is closely related to the occurrence of cancer (Koblinski et al., 2005; Mccabe et al., 2011; Alachkar et al., 2014). The SPARC protein can affect cell cycles, and it inhibits the cell proliferation by EF-hand to binding ${\mathrm{Ca}}^{2+}$ to influence the DNA synthesis (Sage et al., 1995).

SPARC is secreted by the adipocyte, which as a regulator in the extra cellular matrix inhibits fat formation and promotes fibrosis of adipose tissue, which is resistant to insulin. Recently, SPARC has become an important target molecule in the study of diseases such as obesity and diabetes (Harries et al., 2013). According to the research by selecting calf muscle tissue and using RT-PCR to detect the expression of the *SPARC* gene in muscle tissue, it was found that *SPARC* in db/db mouse muscle was highly expressed, suggesting that it may be related to muscle metabolism (Song et al., 2016). Due to its high amino acid homology, SPARC has similar functions in many animals. Therefore, its research on animals is of great significance. In kazak sheep and Tibetan sheep, such as the tail of the adipose tissue of the gene identification, *SPARC* expression may be associated with fat deposition. Sheep tails of excessive fat deposition will increase the investment and the cost of feed, thereby reducing the economic benefits of production (Guo et al., 2018).

SPARC is a kind of acid secreted protein that is rich in cysteine and widely distributed in the body. Because of its special structure of the area, it plays a role in the inhibition of cell cycle, cell adhesion and metastasis, and it has an effect on adjustment between cells and the matrix. At the same time, SPARC also can carry on the mediation to the tissue repair, angiogenesis and other functions (Alkabie et al., 2016). In this research, we found a SNP happens in *SPARC* gene in Chinese cattle breeds. The polymorphism of the mutation was tested in a large group of different Chinese cattle breeds, and the association was analyzed with the growth traits, which would be of benefit to cattle breeding and genetic research.

## 2 Materials and methods

### 2.1 Experimental animals

The animals used in this study were four adult (24 mouth old) female breeds from China: Qinchuan cattle (QC, n=176, Fufeng, Shaanxi Province, China), Pinan cattle (PN, n=136, Xinye County, Henan Province, China), Xianan cattle (XN, n=160, Biyang County, Henan Province, China) and Jiaxian Red cattle (JX, n=144; Jia County, Pingdingshan City, Henan Province). Animal care and study protocols were in accordance with the Animal Care Commission of the College of Veterinary Medicine, Northwest A&F University. Then, the cattle samples were collected from the same breeding farm and the same batch. The body size data of growth traits are accurately measured and recorded according to the same criteria. They are in a state of uniform feeding, and there is no blood relationship between the three generations. And all breeds were feed under the same conditions.

### 2.2 Collection of blood samples and genomic DNA extraction

The samples obtained from the blood belong to each individual. Genomic DNA extracted by phenol chloroform method. The concentration and purity of the genomic DNA were tested and diluted to a uniform final concentration of 25 $\mathrm{ng}\;µL^{-1}$ for subsequent amplification assays.

### 2.3 Collection of different cattle tissues and cDNA extraction

The expression test sample is from XN adult female cattle (n=3). Samples included heart, liver, kidney, lung and muscle tissues. In order to research the relationship between SNP and gene transcription expression level, muscle tissues (n=12) were collected. The total RNA of all tissues was determined using the Trizol method of extraction following the manufacturer's instructions. The cDNA which we collected via RNA was reverse-transcribed using PrimeScript^™^ RT Reagent Kit with gDNA Eraser (Clontech, TaKaRa).

### 2.4 Primer design and amplification assay

SNP primers were designed using the ARMS-primer method (http://primer1.soton.ac.uk/primer1.html, last access: 3 March 2019) based on the DNA sequence of the *SPARC* gene searched in the NCBI (NC_037334.1) and linked to the re-sequencing results. And the RNA expression test primers were designed based on *SPARC* gene mRNA sequence (NM_174464.2). All primer sequences are shown in Table 1.

**Table 1:** Primer information.

	Locus	Primers	Primer sequence (5${}^{\prime }$ to 3${}^{\prime }$)	Genotype pattern (bp)
DNA primers	g.12454T > C	IN-F IN-R out-F out-R	TGGAAGTAGGAGAATTCGATGATGGTTCC CCACCACCTCCTCTTCGGTTTCCGCA CCATCCTCTGTGGGTACCCAAGGCTTT CGTTTCCTTGGGAAGGAACCTCACACAG	168 bp (allele “T”) 136 bp (allele “C”) 306 bp (outer)
mRNA primers	SPARC	Qpcr-F Qpcr-R	ACCATCCTGTGGAACTGCTG CAGGTACCCGTCAATGGGG	113 bp
	β-actin	Qpcr-F Qpcr-R	GTCATCACCATCGGCAATGAG AATGCCGCAGGATTCCATG	84 bp

The same amount of genomic DNA from 50 different individuals of cattle was mixed into a DNA pool for amplification of the gene of interest, and the volume of PCR reaction was 10 µL: the reaction system included 25 ng of genomic DNA (25 $\mathrm{ng}\;µL^{-1}$), 0.5 µL of internal and external primers (10 $\mathrm{pmol}\;µL^{-1}$), 5 µL 2 × Taq PCR Master Mix (GeneStar, Beijing, China), and 2 µL ${\mathrm{ddH}}_{2}O$. The procedure is as follows: the reactants are held at 95 ${}^{\circ }$C for 5 min, at 94 ${}^{\circ }$C for 30 s, down to 60 ${}^{\circ }$C for 30 s, up to 72 ${}^{\circ }$C for 25 s, the above steps for 40 cycles, and finally at 72 ${}^{\circ }$C for 10 min. The PCR product is then sequenced to find mutations. The determination is typically made by sequencing the product in both forward and reverse directions (Sangon, Shanghai, China).

### 2.5 qPCR test of *SPARC* expression level

Fluorescence quantitative detection of *SPARC* expression levels in different tissues was performed using a CFX 96TM real-time quantitative RCR instrument (Bio-Rad, Hercules, CA, USA). The *beta-actin* gene stably expressed in bovine tissues was corrected as an internal reference.

### 2.6 SNP verification and statistical analysis

The SNP phenotype of *SPARC* gene was tested via the T-ARMS-PCR method, and the phenotype showed in 3.0 % agarose gel. The genotype frequency and allele frequency of *SPARC* gene SNPs were statistically analyzed using Excel software. In addition, SPSS V19.0 was used for the correlation analysis of SNP with various growth traits. According to the experimental design, a simplified model processing analysis was performed: Yijk = μ+Tj+Eijk, where Yijk is the individual phenotypic record, μ the population mean, Tj the genotype effect, and Eijk the random error. The *SPARC* gene expression level was quantified by using an optimized method of comparing Ct (ΔΔCt) values (commonly referred to as $2^{-\Delta \Delta \mathrm{Ct}})$. All samples were guaranteed at least three technical replicates, and the intensity ratio ± SD average was obtained therefrom.

## 3 Results

### 3.1 SNP detection and gene phenotype analysis

**Figure 1:**
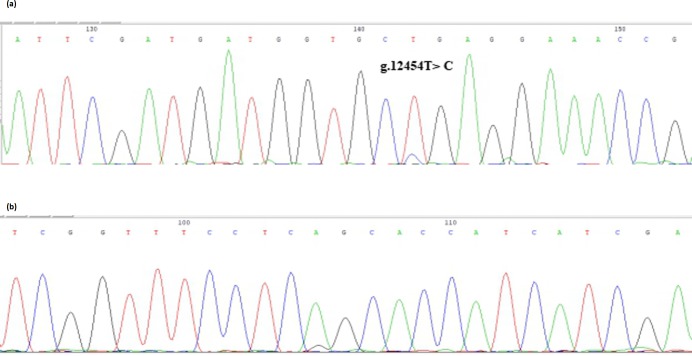
Sequencing of the SNP of cattle *SPARC* gene. Note that the sequence test is the *SPARC* (exon 4) in Chinese cattle DNA pool. **(a)** Forward sequencing; **(b)** reverse sequencing.

**Figure 2:**
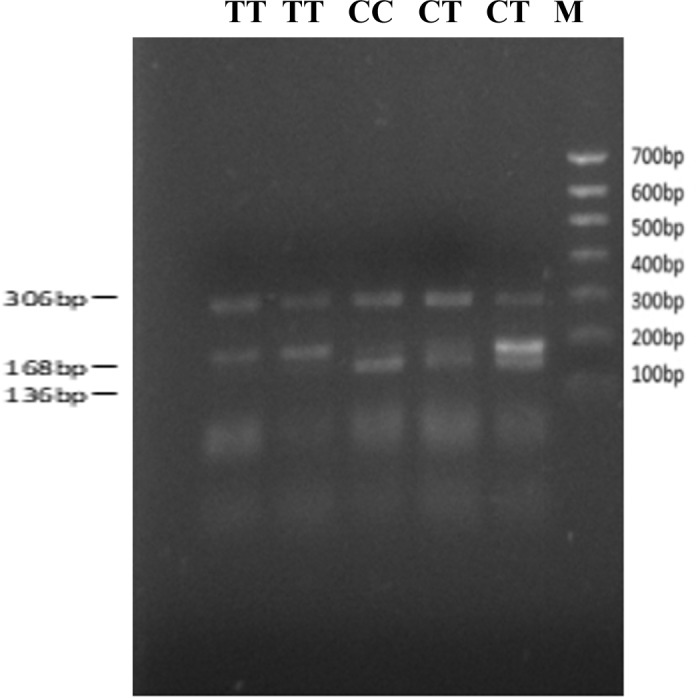
The result of T-ARMS-PCR with SNP: g.12454T > C Note that CC = 306 + 136 bp. TC = 306 + 168 + 136 bp. TT = 306 + 168 bp. M denotes maker II.

The bovine *SPARC* gene was mapped to chromosome 7, and the SNP (g.12454T > C, fourth exon) of the *SPARC* gene was found in the Chinese cattle genome (Fig. 1). The SNP is a synonymous mutation (59Ala > Ala). It is tested by PCR amplification using the ARMS-primer method. Thus, three genotypes were found at the SNP site (g.12454T > C). The length of target band showed in the gel of TT genotype was run into 306 and 168 bp, the length of band of CT genotype was 306, 168 and 136 bp, and the CC genotypic band was 306 and 136 bp (Fig. 2).

### 3.2 Frequency statistics and PIC analysis

Statistical analysis of 616 cattle *SPARC* gene SNP typing found that in QC, PN, XN and JX, the frequency of allele T is much greater than the frequency of allele C. We further calculated He (gene heterozygosity), $N_{e}$ (the number of effective alleles; the reciprocal of homozygotes), Ho (gene homozygous) and PIC (polymorphism information content) with the POPGENE software; the methods are as follows (Nei, 1973; Botstein et al., 1980):

$$\begin{array}{rl} & \mathrm{Ho}=\sum_{i=1}^{n}P_{i}^{2}\;\;\;\;\mathrm{He}=1-\sum_{i=1}^{n}P_{i}^{2}\;\;\;\;N_{e}=1/\sum_{i=1}^{n}P_{i}^{2}\\ & \mathrm{PIC}=1-\sum_{i=1}^{m}\;\;\;\;P_{i}^{2}-\sum_{i=1}^{m-1}\sum_{j=i+1}^{m}2P_{i}^{2}P_{j}^{2}. \end{array}$$

The diversity parameter of PIC is 0.210, 0.325, 0.313 and 0.159 in QC, PN, XN and JX cattle, respectively. The PIC value of QC and JX < 0.25 indicates low genetic diversity. The PIC values of PN and XN > 0.25 and <0.5 indicate intermediate genetic diversity (Table 2).

**Table 2:** Genetic parameters of *SPARC* gene in four cattle populations.

Breeds	Number	Genotypic frequencies			Allelic frequencies		Diversity parameters		
		CC	CT	TT	C	T	He	Ne	PIC
Qinchuan cattle, QC	176	0.050	0.186	0.770	0.138	0.862	0.238	1.312	0.210
Pinan cattle, PN	136	0.020	0.530	0.450	0.287	0.713	0.409	1.965	0.325
Xianan cattle, XN	169	0.020	0.490	0.490	0.263	0.737	0.388	1.634	0.313
Jiaxian red cattle, JX	144	0.010	0.222	0.768	0.112	0.888	0.199	1.248	0.159

Ne: effective allele numbers; He: expected heterozygosity; PIC: polymorphism information content.

### 3.3 The *SPARC* gene transcription level test

**Figure 3:**
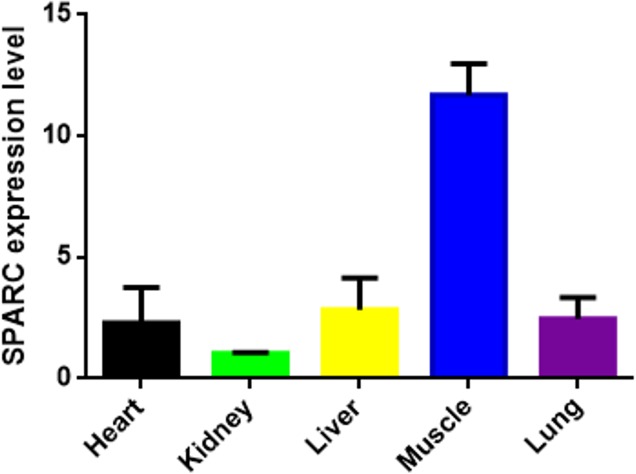
The SPARC mRNA expression level in adult different tissues (relative to the kidney). Note the expression profiling of *SPARC* gene in different tissues in XN cattle. The values are the averages of three independent experiments measured by 2${}^{-\Delta \Delta \mathrm{Ct}}$. Error bars represent the standard deviation (SD) (n=3), and the relative mRNA expression levels of *SPARC* gene are normalized; β-actin was used as an internal reference.

The result showed the transcription level of *SPARC* in five different tissues of adult bovine (Fig. 3). It was expressed in all tissues which we tested (heart, kidney, liver, muscle and lung), and the result showed differences in the tissue expression of the *SPARC* gene in adult cattle. The *SPARC* gene was most highly expressed in muscle tissues compared to others (relative to the kidney). On the contrary, it was most lowly expressed in the kidney compared other tissues.

### 3.4 Correlation analysis of *SPARC* gene SNP and mRNA expression levels

**Figure 4:**
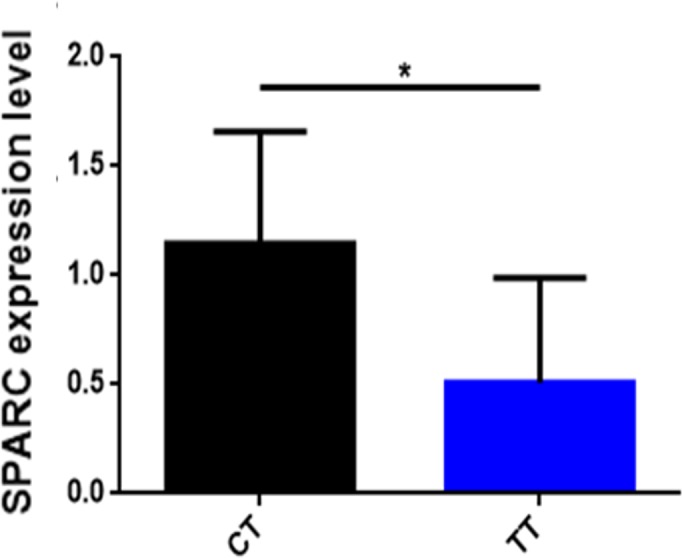
The correlation of SNP with the mRNA expression in *SPARC* gene. Note that three independent experiments were repeated for reliability. An asterisk denotes a significant difference by t test (P<0.05).

To find out whether the influence of SNP affects the mRNA expression level of the *SPARC* gene, we analyzed the correlation of *SPARC* SNP with mRNA expression levels in skeletal muscles from 12 adult cattle (Fig. 4). The results are shown in Fig. 4: the CT gene phenotype (n=6) has a high expression level of the *SPARC* gene compared to the TT gene phenotype (n=6) (p<0.05).

### 3.5 The association result between SNP of *SPARC* gene and phenotypic data

The results show that the SNP in QC and PN cattle has a significant influence on the rump length and hip width (P<0.05); moreover, the mutation also had a significant influence on the rump length and body length of JX cattle (P<0.05). In XN cattle, the SNP has a trend to affect the traits regarding the body height and cannon bone circumference. It shows an effect of CC type > CT type > TT type but not significantly (P>0.05). To summarize, these data results found that the SNP had a positive influence on the growth of skeletal muscle in the hindquarters of cattle, such as body length, hip length and hip width (Table 3).

**Table 3:** Association analysis of SNP with growth traits in four cattle populations.

Breeds	Growth traits	Genotype (mean ± SE)			P value
		CC	CT	TT	
Qinchuan cattle, QC	hip width (cm)	–	41.230±0.669	43.328±0.479	0.016${}^{*}$
	rump length (cm)	–	43.423±0.487	44.689±0.349	0.049${}^{*}$
Pinan cattle, PN	hip width (cm)	$46.000^{b}\pm 1.528$	$46.220^{b}\pm 0.625$	$46.610^{a}\pm 0.472$	0.046${}^{*}$
	rump length (cm)	$45.000^{a}\pm 0.260$	$45.583^{a}\pm 0.540$	$48.537^{a}\pm 0.481$	0.025${}^{*}$
Jiaxian red cattle, JX	body length (cm)	–	139.000±1.801	143.721±1.285	0.047${}^{*}$
	rump length (cm)	–	43.476±1.190	44.882±0.751	0.037${}^{*}$
Xianan cattle, XN	body height (cm)	137.000±1.732	135.688±4.226	134.263±4.722	0.191
	cannon bone circumference (cm)	19.667±1.154	19.372±1.539	18.912±1.254	0.189

Values with different superscripts (a, b) within the same row differ significantly at ${}^{*}P<0.05$.

## 4 Discussion

Secreted protein acidic and rich in cysteine (SPARC), also known as osteonectin or BM-40, is the prototypical matricellular protein, which is secreted by many cells and distributed widely in the body, mainly distributed in the bone, cartilage and eye tissue (Scavelli et al., 2015). The main source of cells in the subcutaneous blood circulation is subcutaneous fat cells, which is a 32 kDa extra cellular matrix glycoprotein. The *SPARC* gene encodes a protein encoded by 298–304 amino acids. The protein has three structurally and functionally distinct modules. One is the amino acid terminal acidic calcium ion-binding region, the I region, which binds to copper ions homologous to follicle-binding elements. The second region is region II, and the third is the extra cellular calcium ion-binding region, region III. Based on such a binding region, it has an antigenic decision, inhibiting endothelial cell proliferation and angiogenesis. It also has functions such as decellularization (Salvatierra et al., 2015; Wong and Sukkar, 2016).

However, there are few reports on the genes of animals, especially cattle. According to our study, a SNP site of loci in *SPARC* gene is significantly associated with cattle body size. It was found to be significantly associated with character reflecting the development of the hindquarters of the cattle. The polymorphism statistics showed that the polymorphism of the mutation in the hybrid cattle of XN cattle and PN cattle was more polymorphic than that of the Chinese local cattle breed, which may be related to hybridization, and the mutation is suitable for molecular markers of yellow cattle breeding. The *SPARC* gene was highly expressed in skeletal muscle tissue. Although this mutation occurs on the fourth exon but belongs to a synonymous mutation (59Ala > Ala), it does not alter the protein coding. However, studies have also reported that synonymous mutations have a protein regulation function effect on the phenotype, etc., through other complex methods (Nackley et al., 2006; Kimchi-Sarfaty et al., 2007; Sauna and Kimchi-Sarfaty, 2011). And we also find that the SNP of the *SPARC* gene may change its expression in skeletal muscle tissue. Therefore, it is necessary to further study how the occurrence of this mutation affects the growth traits of cattle through more in-depth individual cell tests.

## 5 Conclusion

In this study, a SNP locus of *SPARC* gene was obtained by analyzing the mutation site of the *SPARC* gene. Among the four varieties tested, the traits of Qinchuan cattle, Pinan cattle and Jiaxian red cattle and the mutation of *SPARC* gene have a significant relationship (p<0.05). This mutation is of positive significance for early selection and breeding, and it provides new research directions and ideas.

## Data Availability

The original data are available upon request to the corresponding author.

## References

[bib1.bib1] Alachkar H, Santhanam R, Maharry K, Metzeler KH, Huang XM, Kohlschmidt J, Mendler JH, Benito JM, Hickey C, Neviani P, Dorrance AM, Anghelina M, Khalife J, Tarighat SS, Volinia S, Whitman SP, Paschka P, Hoellerbauer P, Wu YZ, Han L, Bolon BN, Blum W, Mrózek K, Carroll AJ, Perrotti D, Andreeff M, Caligiuri MA, Konopleva A, Garzon R, Bloomfield CD, Marcucci G (2014). *SPARC* promotes leukemic cell growth and predicts acute myeloid leukemia outcome. J Clin Invest.

[bib1.bib2] Alkabie S, Basivireddy J, Zhou LX, Roskams J, Rieckmann P, Quandt JA (2016). *SPARC* expression by cerebral microvascular endothelial cells in vitro and its influence on blood-brain barrier properties. J Neuroinflamm.

[bib1.bib3] Botstein D, White RL, Skolnick M, Davis RW (1980). Construction of a genetic linkage map in man using restriction fragment length polymorphisms. Am J Hum Genet.

[bib1.bib4] Carignano HA, Roldan DL, Beribe MJ, Raschia MA, Amadio A, Nani JP, Gutierrez G, Alvarez I, Trono K, Poil MA, Miretti MM (2018). Genome-wide scan for commons snps affecting bovine leukemia virus infection level in dairy cattle. BMC Genomics.

[bib1.bib5] Dang YL, Li MX, Yang MJ, Cao XK, Lan XY, Lei CZ, Zhang CL, Lin Q, Chen H (2014). Identification of bovine NPC1 gene cSNPs and their effects on body size traits of Qinchuan cattle. Gene.

[bib1.bib6] Guo JZ, Tao HX, Li PF, Li L, Zhong T, Wang LJ, Ma JY, Chen XY, Song TZ, Zhang HP (2018). Whole-genome sequencing reveals selection signatures associated with important traits in six goat breeds. Sci Rep-UK.

[bib1.bib7] Hamajima N, Saito T, Matsuo K, Kozaki K, Takahashi T, Tajima K (2000). Polymerase chain reaction with confronting two-pair primers for polymorphism genotyping. Cancer Sci.

[bib1.bib8] Harries LW, McCulloch LJ, Holley JE, Rawling TJ, Welters HJ, Kos K (2013). A role for *SPARC* in the moderation of human insulin secretion. PloS one.

[bib1.bib9] Kimchi-Sarfaty C, Oh JM, Kim IW, Sauna ZE, Calcagno AM, Ambudkar SV, Gottesman MM (2007). A “silent” polymorphism in the MDR1 gene changes substrate specificity. Science.

[bib1.bib10] Koblinski JE, Kaplansinger BR, Vanosdol SJ, Wu M, Engbring JA, Wang S, Goldsmith CM, Piper JT, Vostal JG, Harms JF, Welch DR, Kleinman HK (2005). Endogenous osteonectin/sparc/bm-40 expression inhibits mda-mb-231 breast cancer cell metastasis. Cancer Res.

[bib1.bib11] Mccabe NP, Kerr BA, Madajka M, Vasanji A, Byzova TV (2011). Augmented osteolysis in sparc-deficient mice with bone-residing prostate cancer. Neoplasia.

[bib1.bib12] Moravčíková N, Trakovická A, Navrátilová A, Nádaský R (2018). Associations between snps in bovine estrogen receptor gene and production traits in holstein cattle. J Microbiol Biotechn.

[bib1.bib13] Nackley AG, Shabalina SA, Tchivileva IE, Satterfield K, Korchynskyi O, Makarov SS, Maixner W, Diatchenko L (2006). Human catechol-O-methyltransferase haplotypes modulate protein expression by altering mRNA secondary structure. Science.

[bib1.bib14] Nakajima A, Kawaguchi F, Uemoto Y, Fukushima M, Yoshida E, Iwamoto E, Akiyama T, Kohama N, Kobayashi E, Honda T, Oyama K, Mannen H, Sasazaki S (2018). A genome-wide association study for fat-related traits computed by image analysis in japanese black cattle. Anim Sci J.

[bib1.bib15] Nei M (1973). Analysis of gene diversity in subdivided populations. P Natl Acad Sci USA.

[bib1.bib16] Sage EH, Bassuk JA, Yost JC, Folkman MJ, Lane TF (1995). Inhibition of endothelial cell proliferation by SPARC is mediated through a ${\mathrm{Ca}}^{2+}$-binding ef-hand sequence. J Cell Biochem.

[bib1.bib17] Salvatierra E, Alvarez MJ, Leishman CC, Baquero ER, Lutzky VP, Chuluyan HE, Podhajcer OL (2015). *SPARC* Controls Melanoma Cell Plasticity through Rac1. PloS one.

[bib1.bib18] Sauna ZE, Kimchi-Sarfaty C (2011). Understanding the contribution of synonymous mutations to human disease. Nat Rev Genet.

[bib1.bib19] Scavelli K, Chatterjee A, Rhee DJ (2015). Secreted Protein Acidic and Rich in Cysteine in Ocular Tissue. J Ocul Pharmacol Th.

[bib1.bib20] Song HY, Yang XY, Lei D (2016). Increased SPARC expression in skeletal muscle and adipose tissue of db/db mice. Int J Clin Exp Patho.

[bib1.bib21] Termine JD, Kleinman HK, Whitson SW, Conn KM, McGarvey ML, Martin GR (1981). Osteonectin, a bone-specific protein linking mineral to collagen. Cell.

[bib1.bib22] Wong SL, Sukkar MB (2016). The *SPARC* protein: an overview of its role in lung cancer and pulmonary fibrosis and its potential role in chronic airways disease. Brit J Pharmacol.

[bib1.bib23] Workman G, Bradshaw AD (2017). Production and purification of recombinant human *SPARC*. Method Cell Biol.

